# Emulsion-Based Postbiotic Formulation Is Comparable to Viable Cells in Eliciting a Localized Immune Response in Dairy Cows With Chronic Mastitis

**DOI:** 10.3389/fmicb.2022.759649

**Published:** 2022-03-22

**Authors:** Harsh Mathur, Kevin Linehan, James Flynn, Noel Byrne, Pat Dillon, Muireann Conneely, Ghjuvan Grimaud, Colin Hill, Catherine Stanton, R. Paul Ross

**Affiliations:** ^1^Teagasc Food Research Centre, Moorepark, Fermoy, Ireland; ^2^APC Microbiome Ireland, University College Cork, Cork, Ireland; ^3^School of Microbiology, University College Cork, Cork, Ireland; ^4^Dairy Production Research Centre, Teagasc, Moorepark, Fermoy, Ireland; ^5^Teagasc Animal and Grassland Research and Innovation Centre, Moorepark, Fermoy, Ireland

**Keywords:** mastitis, emulsion, lacticin 3147, somatic cell counts, live bio-therapeutic, probiotics, postbiotic

## Abstract

Bovine mastitis is a disease with a multi-etiological nature, defined as an infection and inflammation of the udder. Mastitis represents a significant ongoing concern in the dairy industry, leading to substantial losses in profits and revenue for farmers worldwide. The predominant causes of bovine mastitis include the pathogens *Staphylococcus aureus*, *Streptococcus dysgalactiae*, *Streptococcus uberis*, and *Escherichia coli*. Antibiotic administration is currently the main treatment option for mastitis. However, there is a pressing need for alternative therapies to treat and prevent the disease, especially with the emergence of antibiotic-resistant, mastitis-causing pathogens, resulting in antibiotic treatment failure. One such example is live bio-therapeutics (also known as probiotics), such as *Lactococcus lactis* DPC3147. The efficacy of this live bio-therapeutic has been demonstrated in several previous trials by our group. The most recent of these trials showed that an emulsion-based formulation of this strain was as effective as a commercial antibiotic formulation in treating sub-clinical and clinical cases of bovine mastitis. Here, we report the results of a follow-up field trial, in which we sought to gain insight into the mechanism of action of such live bio-therapeutics, focussing on chronic mastitis cases. We treated 28 cows with chronic mastitis with two separate emulsion-based formulations containing either viable *L. lactis* DPC3147 cells (15 cows) or heat-killed *L. lactis* DPC3147 cells (13 cows). We then evaluated the efficacies of the two formulations (two treatment groups) in terms of stimulating a localized immune response (quantified by measuring IL-8 concentrations in milk collected from udders affected by mastitis) and efficacies in terms of cure rates (quantified by reductions in somatic cell counts and absence of pathogens). We demonstrate that the presence of heat-inactivated bacteria (a postbiotic) was as effective as the live bio-therapeutic in eliciting a localized immune response in cows with chronic mastitis. The response to heat-killed cells (postbiotic) reported herein could have beneficial implications for farmers with regard to prolonging the shelf life of such emulsion-based formulations containing heat-killed cells of *L. lactis* DPC3147 for curing cows with mastitis.

## Introduction

Milk is amongst the most versatile products in the food industry. World milk production was recorded at 823 million tons in 2017 and it is expected to increase by 22% by 2027 (Organization for Economic Co-operation and Development, 2018). In addition to economic importance, milk and dairy products are important sources of macro and micronutrients in the human diet (Muehlhoff et al., 2013), including high-quality proteins, fatty acids, calcium, potassium, phosphorus, vitamin D, riboflavin, and vitamin B12 (Keast et al., 2013; O’Neil et al., 2018). Mastitis is the most prevalent and economically significant disease in dairy cattle worldwide (Castelani et al., 2019), primarily due to depleted milk production, discarded milk, premature culling, and treatment costs (Halasa et al., 2007; Bar et al., 2008; Hertl et al., 2010). In addition to negative impacts on milk production and quality (Detilleux et al., 2015), mastitis also influences the reproductive system of the animal (Kumar et al., 2017) and increases susceptibility to other diseases (Cha et al., 2013). Thus, decreasing the rates of mastitis and finding effective treatment options is crucial for ensuring a healthier herd, minimizing high somatic cell counts (SCCs) in cows, maximizing production yields and profits. The conventional treatment options for mastitis usually involve the use of antibiotics, such as pirlimycin, methicillin, cloxacillin, amoxicillin, novobiocin, penicillin G, dihydrostreptomycin, cephapirin, and erythromycin, which are all approved for bovine mastitis treatment and prevention (Nader-Macías et al., 2011). The principal etiological agents of bovine mastitis are *Staphylococcus aureus*, *Streptococcus dysgalactiae*, and *Streptococcus uberis* (Crispie et al., 2008; Zadoks et al., 2011; Abebe et al., 2016; Bi et al., 2016). While antibiotics have proven to be effective in some cases against mastitis, a plethora of studies indicate that mastitis-causing pathogens are becoming resistant to antibiotics and the cure rates of conventional treatments are low (Freitas et al., 2018). Furthermore, mastitis caused by *S. aureus* can be recalcitrant to antibiotics due to its ability to evade the innate and adaptive immune response of the cow (Barkema et al., 2006; Zadoks and Schukken, 2006; Piotr et al., 2013; Zaatout et al., 2020), lending credence to the concern that antibiotics are no longer considered to be sufficient against *S. aureus*-induced bovine mastitis (Pietrocola et al., 2017).

The emergence of antimicrobial resistance (AMR) due to the overuse of antibiotics in food-producing animals has become a major concern (Xiong et al., 2018), especially with respect to the risk of developing newly resistant bacteria that could be transmitted from animals to humans (Foutz et al., 2018). It is therefore imperative that preventative and alternative treatment plans are devised to reduce reliance on antibiotics in the dairy herd (McEwen and Collignon, 2018). To date, other forms of therapies (or combinations of antimicrobials) for mastitis have been reported with varying levels of efficacy (Angelopoulou et al., 2019; Cheng and Han, 2020). Bacteriocins such as nisin A have demonstrated antibacterial activity against mastitis pathogens (Malvisi et al., 2016). Immune stimulants such as ginseng (Beccaria et al., 2018) and the use of plant-derived compounds (Maia et al., 2018) and immune proteins such as lactoferrin (O’Halloran et al., 2016) have also been tested in this regard. Probiotic lactic acid bacteria (LAB) strains have been reported to eliminate mastitis-causing staphylococcal biofilms (Wallis et al., 2019). Furthermore, *Lactobacillus casei* BL23 has demonstrated anti-inflammatory properties on *S. aureus*-stimulated bovine mammary epithelial cells (Souza et al., 2018). However, there remains much mechanistic and trial work to elucidate the potential of probiotics as substitutes for antibiotics. Finally, phage therapy for treating bovine mastitis has been hampered on account of the inhibition of phages by compounds present in bovine milk (O’Flaherty et al., 2005). Phage endolysins, however, represent promising antimicrobial alternatives with proven potent activity against mastitis-causing pathogens (Zhou et al., 2017). However, endolysins require genetic engineering for both production and design, suggesting these are drugs of the future (Angelopoulou et al., 2019).

Our group has shown that the two-component bacteriocin produced by the LAB *L. lactis* DPC3147 inhibits a broad range of Gram-positive mastitis pathogens (Ryan et al., 1998, 1999; Klostermann et al., 2008, 2010). *Lactococcus lactis* strains are routinely used as dairy starter organisms and numerous strains have been granted GRAS (generally regarded as safe) status in the dairy industry. Studies by Ryan et al. (1999) and Twomey et al. (2000), reported that lacticin 3147 combined with a bismuth-based teat seal prevented *Strep. dysgalactiae* infections in dry cows (Ryan et al., 1999) and *S. aureus* infections in lactating cows (Twomey et al., 2000). In a separate field trial, Klostermann et al. (2008) evaluated the bactericidal activity of a freeze-dried preparation of the lacticin 3147-producing culture resuspended in sterile water and found that the treatment was as efficacious as conventional antibiotic treatment. Furthermore, that study noted that a Gram-negative, lacticin 3147-insensitive *E. coli* strain was also one of the mastitis-causing pathogens that was eliminated by this freeze-dried preparation. Thus, it has been suggested that a mechanism other than bacteriocin production was contributing to the elimination of this Gram-negative pathogen (Klostermann et al., 2008). Additionally, Crispie et al. (2008) reported that infusion with freeze-dried *L. lactis* DPC3147 rapidly stimulated the host intra-mammary immune system leading to the recruitment of lymphocytes and polymorphonuclear leukocytes (PMNs), to the mammary gland along with the localized production of acute-phase proteins (APP). A combination of these factors contributes to clear the mammary gland of the offending pathogen (Crispie et al., 2008). In a separate study (Klostermann et al., 2010), it was reported that a teat dip containing lacticin 3147 was efficacious at eliminating the mastitis pathogens *S. aureus*, *Strep. dysgalactiae* and *Strep. uberis* from cows’ teats. However, producing sufficient quantities of pure antimicrobial peptides would be costly and is one of the main bottlenecks precluding the use of purified antimicrobial bacteriocins to treat bovine mastitis. More recently, our group developed a novel, live bio-therapeutic formulation, containing a liquid paraffin-based emulsion of *L. lactis* DPC3147. This live bio-therapeutic formulation displayed a 47% cure rate compared to a 50% cure rate for the routinely used commercial antibiotic formulation Terrexine™ for the treatment of cows with clinical and sub-clinical mastitis (Kitching et al., 2019).

The time-effective and inexpensive nature of producing this emulsion-based live bio-therapeutic formulation, compounded by its prolonged shelf life compared to previous aqueous-based formulations of the same, suggest that this product may represent a realistic alternative therapeutic option for bovine mastitis. In addition, the single-dose administration of this live bio-therapeutic formulation is a further advantage, as it can hasten the return of the milk to the milk pool and minimize or prevent withholding of milk from processing for several days, in comparison to some commercial antibiotics. The primary objective of this current trial was to investigate if such recently developed emulsion-based formulations derived from *L. lactis* DPC3147 need to contain viable cells or whether heat-inactivated cells thereof can elicit an equally potent localized immune response in cows with chronic mastitis. The outcome of this field trial reported herein demonstrates that a formulation containing heat-killed cells of *L. lactis* DPC3147 is equally efficacious at eliciting a localized interleukin 8 (IL-8) response upon infusion of udders affected by mastitis. The equally potent immuno-stimulatory effects of emulsion-based formulations containing heat-killed cells of *L. lactis* DPC3147 could present farmers with additional alternative therapeutic options with a prolonged shelf life, for treating cows with mastitis.

## Materials and Methods

### Preparation of Liquid Paraffin-Based Emulsions Containing Viable and Heat-Killed *Lactococcus lactis* DPC3147 Cells

The emulsion-based formulations were prepared as described by Kitching et al. (2019), with minor modifications. Briefly, liquid paraffin and polysorbate 80 were both purchased from Sigma-Aldrich (Vale Road, Arklow, Ireland). Polysorbate 80 (10%) was prepared in sterile autoclaved DNAase and RNAase-free water and sterilized by filtering twice through 0.2 μm syringe filters. Forty-five milliliters of liquid paraffin were poured into 250 ml glass beakers and sterilized under dry heat at 170°C for 2 h. This was subsequently allowed to cool to room temperature prior to use. Prior to preparation of the emulsion, the blade of an Ultra-Turrax® homogenizer was autoclaved and then steeped in 1% v/v Chlorus and UV-sterilized under laminar airflow for 1 h.

Ten milliliter overnight cultures of *L. lactis* DPC3147 were grown for 16 h at 30°C in LM17 (containing 0.5% lactose) broth (Merck). The overnight cultures were subsequently centrifuged at 8,000*g* for 15 min at 4°C using a Thermo Scientific Sorvall centrifuge to isolate the cell pellet. The cell pellets were washed twice with 10 ml of sterile autoclaved RNAase-free water between centrifugation steps. Finally, the cells were resuspended in 15 ml of 10% polysorbate 80. Forty-five milliliters of sterile liquid paraffin were homogenized with 15 ml of the 10% polysorbate 80 (mixed with cells) at 10,000 rpm in an Ultra-Turrax® homogenizer for 5 min under ice to yield a final cell concentration of approximately 1 × 10^9^ cfu per 5 ml dose (equivalent to approximately 2 × 10^8^ cfu/ml) in the emulsion. *Lactococcus lactis* DPC3147 cells resuspended in 10% polysorbate 80 were slowly poured into the liquid paraffin during homogenization. Thus, the emulsion contained a final concentration of 2.5% polysorbate 80. To prepare heat-killed *L. lactis* DPC3147 cells, the above-mentioned steps were followed identically. However, just prior to resuspending the cells in 10% polysorbate 80, the cells were resuspended in 1 ml of sterile water and heat-treated in a boiling water bath at 100°C for 12 min. After boiling, the heat-killed cells were cooled to room temperature before resuspending them in 14 ml of 10% polysorbate 80. Homogenization steps were conducted with the Ultra-Turrax® as described above.

The number of viable cells was quantified in the emulsion formulations in terms of colony forming units/ml (cfu/ml) by conducting viable plate counts. Maximum recovery diluent (MRD, Oxoid, Basingstoke, Hampshire, United Kingdom) was utilized as a diluent in all cases. *Lactococcus lactis* DPC3147 (and heat-killed versions thereof) were plated onto LM17 agar. Plates were incubated for 24 h at 30°C and cfu/ml determined. Agar plates from heat-treated cells were incubated for up to 72 h at 30°C to ensure that all the cells were killed as confirmed by the absence of any colonies on agar plates after 72 h of incubation. While we did not check bacterial cell integrity after heat inactivation by microscopy, we concluded that the absence of any colonies whatsoever, even after a prolonged incubation of 72 h meant that our heat-killing procedure was 100% effective and no bacterial cells were able to recover and form colonies on agar plates.

### Cows Selected for the Emulsion Formulation Trial, Intra-Mammary Infusion, and Sampling

This field trial was approved by the Teagasc Animal Ethics Committee (Approval number TAEC186-2018) and by the Health Products Regulatory Authority (HPRA; Project Authorization number AE19132/P086). Holstein-Friesian dairy cows with mastitis were first identified in the herd using bulk cow somatic cell counts (SCCs) and only animals with a SCC >250,000 cells/ml were selected for the trial. Once cows were selected on the basis of these bulk cow SCCs, quarter sampling was conducted to identify the infected udder and quarter sample SCCs were quantified thereafter. Cows with clinical mastitis displayed obvious signs of udder inflammation and/or general malaise, clotted/abnormal milk production as well as the presence of pathogens in milk. G*Power calculations prior to conducting the trial revealed that *n* = 13 cows for each of the two treatment groups were sufficient for statistically valid results. However, this number was rounded off to *n* = 15 cows per treatment group to account for possible dropouts. To assess the response to each of the treatment groups, cows that were presenting with high SCC were identified in the herd and the affected quarter was infused at random with a one-time dose of emulsion preparation containing any one of the two formulations, which were made fresh (approximately 1 × 10^9^ cfu of *L. lactis* DPC3147 or heat-killed versions thereof, per 5 ml dose; equivalent to approximately 2 × 10^8^ cfu/ml; *n* = maximum of 15 cows per treatment group). The selection of the treatment group was at random, to prevent bias and variability in the results. In specific cases where a cure was successful (defined by a reduction in SCC counts from initially high numbers to between 250,000 and 750,000 cells/ml and absence of pathogens upon plating within 5–7 days post-infusion), antibiotics were not required at all. However, in specific cases where there was treatment failure as a result of our formulations even after 7 days (and/or the cows were unable to mount a potent immune response naturally to result in a natural cure), conventional antibiotic intervention took place 7 days post-infusion to cure the cows, as per good veterinary and clinical practice. Any such cows were administered two doses of a commercial antibiotic. The commercial dual antibiotic formulation consists of cephalexin (200 mg) and kanamycin (100,000 I.U.) and is routinely utilized to treat lactating cows with clinical or sub-clinical mastitis in the Moorepark herd at the Teagasc Animal and Grassland Research and Innovation Centre, Moorepark, Ireland. The cows were considered still infected if the SCC was still high 7 days post-infusion, as the main criterion (and such cases of high SCCs were routinely validated by plating for pathogens as well, even after the conclusion of our investigative trial with our two treatment groups described here in this study).

All the treatments from each of the two treatment groups were directly administered into the teat sinus as previously described with minor modifications (Crispie et al., 2008; Klostermann et al., 2008). Briefly, the teat in question was swabbed with 70% v/v ethanol on cotton wool, prior to sampling. A milk sample from the affected quarter was collected just before infusion. After this, 5 ml of an emulsion-based formulation from one of the two treatment groups, containing approximately 1 × 10^9^ cfu (or heat-killed versions thereof) per 5 ml dose (equivalent to approximately 2 × 10^8^ cfu/ml) was infused into the mammary gland using sterile blunt-ended steel syringes (17 mm in length). Milk samples were taken at time 0 (just prior to infusion), 6 h post-infusion and subsequently at intervals up to 7 days post-infusion for analysis of SCC, interleukin (IL)-8 titres and the presence of mastitis-causing pathogens was detected by plating each of these milk samples on Blood agar (Oxoid Ltd., Basingstoke, Hampshire, United Kingdom). The respective pathogens were identified as follows: *S. aureus* produced creamy, greyish-white and occasionally golden-yellow colonies on blood agar, 3–5 mm in diameter with typical zones of haemolysis. Streptococci produced small, smooth, translucent, cone shaped colonies on blood agar. *Strep. dysgalactiae* colonies produced characteristic narrow zones of pale green discoloration of the medium accompanied usually by a narrow zone of haemolysis. *Strep. uberis* colonies caused a diffuse browning of the aesculin blood agar. *Escherichia coli* produced large (3–4 mm) grey colonies. Such *E. coli* colonies can sometimes also show haemolysis and frequently produce mucoid colonies.

Overall, the cows with high bulk SCCs (and subsequently identified high quarter SCCs as briefly described above) as part of this trial were selected at random to prevent bias. The cows selected had a mixture of lactation ranks, ranging from ranks 1 to 7. Therefore, the cows were selected at random from early, mid and late lactation stages, as part of this trial to prevent bias. Upon retrospective assessments of the historical records of the cows, we concluded that the majority of cows had suffered from previous episodes of mastitis. Aside from the Moorepark herd (from where most of the cows were selected), there was one other nearby farm (Curtins farm) from which cows were selected as well. The selection of cows took place in the time period between November 2018 and July 2019.

### Somatic Cell Count Measurements

SCC/ml of milk samples were determined as previously described with a few minor modifications (Klostermann et al., 2008, 2010). Briefly, milk samples were collected from the relevant quarters at time 0 (just before treatment) and at designated time points post-treatment, as described above. SCC/ml in raw milk were measured using a Somacount 300® (Bentley Instruments Incorporated, United States).

### Determination of IL-8 Titres in Milk Samples Using ELISA

Milk samples were collected as described above and centrifuged for 30 min at 44,000*g*, in order to remove the fat layer from the milk using a sterile pipette tip. After centrifugation, the supernatants from the milk samples were subjected to IL-8 analysis by ELISA or samples were frozen at −80°C for analysis at a later stage, as described previously (Kitching et al., 2019). The bovine IL-8 (CXCL8) ELISA development kit (Mabtech AB, Nacka Strand, Sweden) was utilized to quantify IL-8 titres in milk samples collected at different time points pre- and post-treatment, from cows treated with emulsion preparations containing one of the two treatment group formulations. Briefly, the following protocol was used: A 96-well Nunc-Immuno plate (Thermo Scientific, Ballycoolen, Dublin, Ireland) was pre-coated with 100 μl of mAb MT8H6 at a concentration of 2 μg/ml (diluted in PBS pH 7.4) and the plate was stored at 4°C for 16 h. After 16 h, all wells were washed twice with 200 μl phosphate-buffered saline (PBS pH 7.4). The plate was subsequently blocked with 200 μl PBS (supplemented with 0.05% Tween 20 and 0.1% bovine serum albumin) and the plate was covered in tin foil and kept at room temperature for 1 h. After 1 h, the plate was washed five times using PBS supplemented with 0.05% Tween 20 (hereafter referred to as wash buffer). After this, 100 μl of IL-8 standards ranging from concentrations of 6.25 to 1,600 pg/ml were added to the wells in triplicate, as per the manufacturer’s instructions. Milk samples collected at different time points were diluted 1:10, and 1:100 in PBS (pH7.4) and 100 μl of the undiluted samples, 1:10 dilutions and 1:100 dilutions were added to the wells in the microtiter plate in triplicate. The plate was covered in tin foil and incubated for 2 h at room temperature. After 2 h, each of the wells was washed with wash buffer five times. After these wash steps, 100 μl of mAb 26E5-biotin at a final concentration of 0.1 μg/ml was added to each well and the plate covered in tin foil and incubated for 1 h. As before, the wells were washed five times with wash buffer after the 1 h incubation step. One hundred microliters of Streptavidin-ALP diluted 1:1000 was added to each well and the plate covered in tin foil and incubated for 1 h at room temperature, followed by washing five times. Finally, phosphatase substrate (Sigma-Aldrich, Vale Road, Arklow, Ireland) at a final concentration of 1 mg/ml was prepared using sterile de-ionized water supplemented with 7.5 mg/ml glycine, 1 mM ZnCl_2_ and 1 mM MgCl_2_ and the pH was adjusted to pH 10.4 using 2 M NaOH. One hundred microliters of this substrate solution was added to each of the wells and the plate covered in tin foil and incubated for 1 h at 37°C. A plate reader (Synergy HT, BioTek) was used to take absorbance readings at 405 nm. Graph Pad Prism software (version v9.1.0) with five-parameter logistics was used to interpolate IL-8 values. However, in the rare event that five-parameter logistics failed to interpolate IL-8 values for certain quarters, the less stringent four-parameter logistics was utilized instead of five-parameter logistics in these specific cases.

### Statistical Analyses

Statistical analyses described herein were performed in Graph Pad Prism v9.1.0. Comparison of the cure rate of the two treatment groups was performed using a Fisher’s exact test. IL-8 and SCC data were first tested for normality using the Shapiro–Wilk test. Non-parametric Wilcoxon tests were used to evaluate statistical differences between heat-killed *L. lactis* DPC3147 and viable *L. lactis* DPC3147 treatment groups in all cases. A spline-based statistical tool was also used to compare the whole time series (R package splinectomeR; Shields-Cutler et al., 2018). A difference was considered statistically significant if *p* < 0.05 in all cases.

To ordinate the sample, we first calculated dissimilarity matrices from the cows’ time series using the “vegdist” function from the “vegan” R package (Bray–Curtis distances). Dissimilarity matrices were subjected to classical multidimensional scaling (PCoA) to obtain the first two principal coordinates, as well as the variance explained by each, using the “pcoa” function from the “ape” R package. The dissimilarity matrices used for PCoA were also used for permutational multivariate analysis of variance (PERMANOVA; “adonis2” function from “vegan”). Pairwise PERMANOVA were calculated using the “pairwiseAdonis” R package, and value of *p* were adjusted using the Bonferroni method. A difference was considered statistically significant if the adjusted *p* < 0.05. We finally calculated the Spearman correlation coefficients between SCC and IL-8 using the “cor.test” function from “stats” R package.

## Results

### *Lactococcus lactis* Cells Do Not Need to Be Viable to Elicit a Localized IL-8 Response

**Figure 1:**
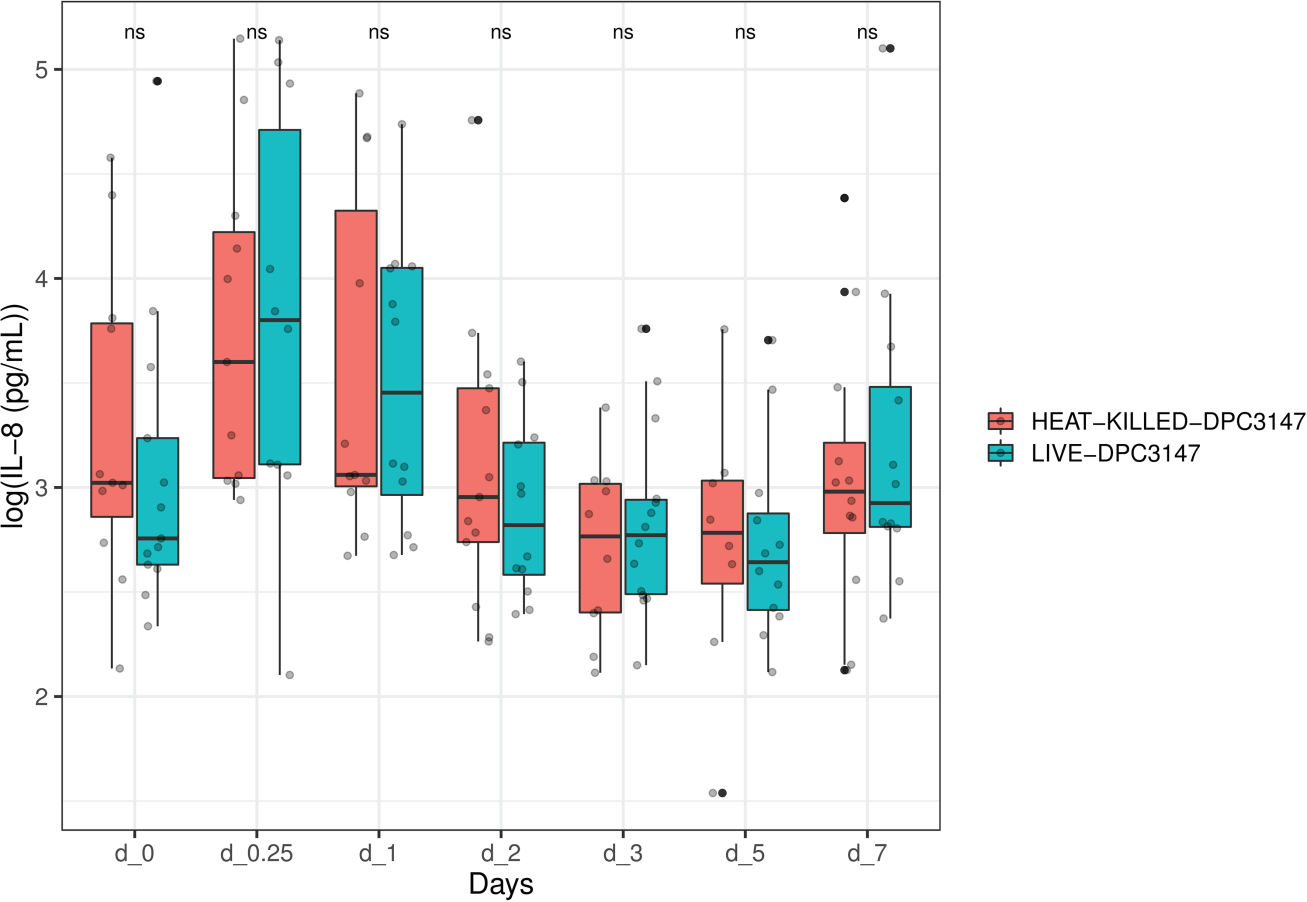
A non-parametric Wilcoxon test was used to compare the IL-8 titres elicited by viable *Lactococcus lactis* DPC3147 cells versus heat-killed DPC3147 cells. The figure shows that there was no significant difference in IL-8 responses in cows treated with either viable DPC3147 cells or heat-killed DPC3147 cells at any of the time points (*p* > 0.05). This indicates that heat-killed cells can elicit an equally potent IL-8 response as viable DPC3147 cells.

**Figure 2:**
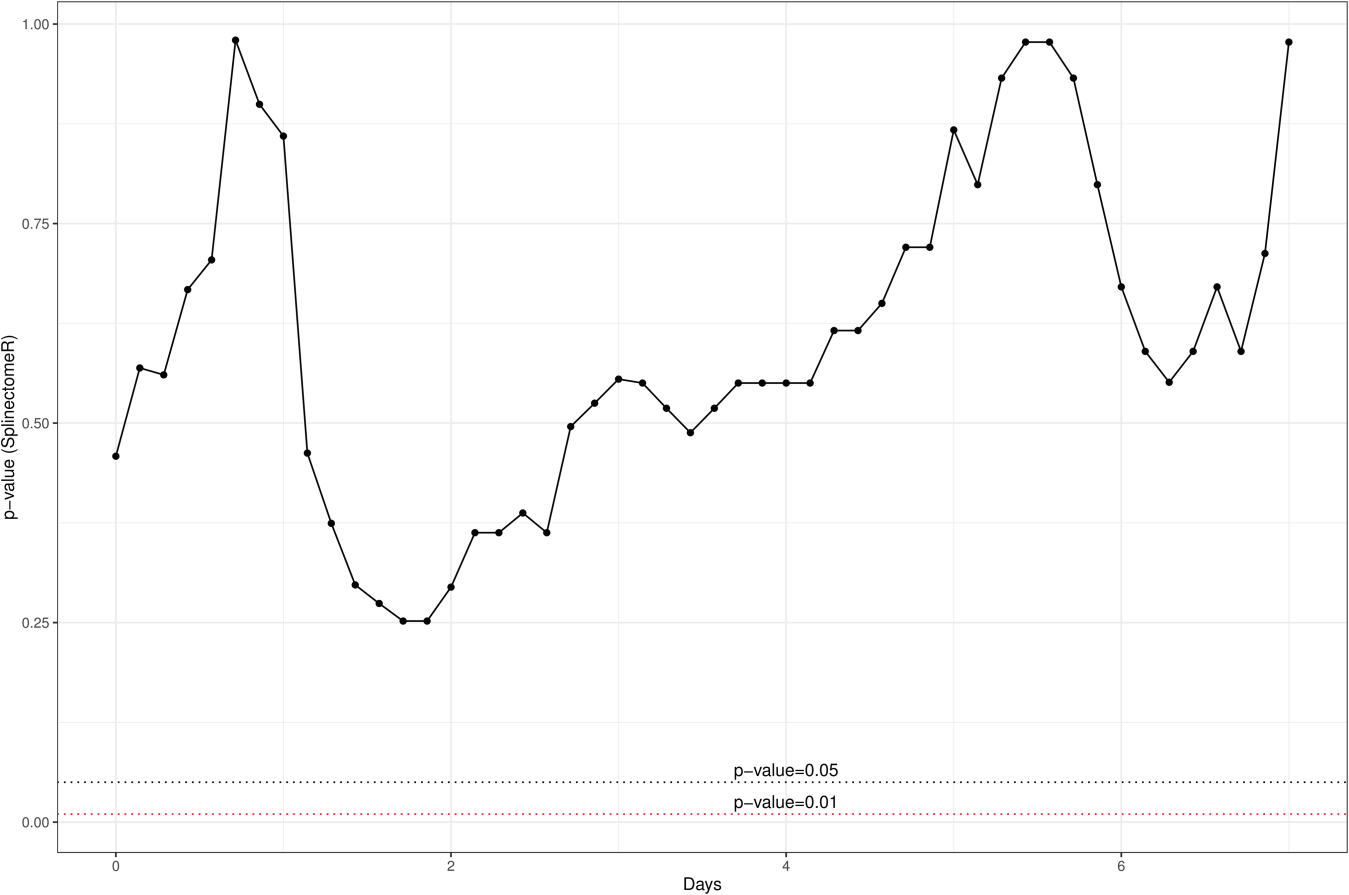
A spline-based statistical tool was used to compare the value of *p* of IL-8 titres in the whole time series (R package splinectomeR; Shields-Cutler et al., 2018). The differences between the two treatment groups are not significant for IL-8 (*p* = 0.93), further highlighting that heat-killed cells have the ability to elicit an equally potent localized IL-8 response as do viable DPC3147 cells.

We opted to quantify the localized IL-8 response upon infusion of both treatment options as previous field trials of this nature in our group had indicated that IL-8 was the main cytokine involved in a localized inflammatory response and responsible for the recruitment of polymorphonuclear leucocytes and neutrophils to the site of infection (Beecher et al., 2009). While there was a large inter-animal variation of IL-8 concentrations in this trial as well, overall, it was apparent that heat-killed *L. lactis* DPC3147 cells elicited an equally potent IL-8 response as viable *L. lactis* DPC3147 cells (Figure 1). A Shapiro test of normality was initially used to show that the IL-8 values were not normally distributed (*p* < 0.05) and therefore, a non-parametric Wilcoxon test was used to compare the IL-8 titres elicited by viable *L. lactis* DPC3147 cells versus heat-killed DPC3147 cells. Figure 1 shows that there was no significant difference in IL-8 responses in cows treated with either viable DPC3147 cells or heat-killed DPC3147 cells at any of the time points (*p* > 0.05). This indicates that formulations containing heat-killed cells were able to trigger an equally potent IL-8 response as formulations containing viable DPC3147 cells. In addition, a spline-based statistical tool was used to compare the value of *p* of IL-8 values in the whole time series (R package splinectomeR; Shields-Cutler et al., 2018; Figure 2). As shown in Figure 1, the differences in IL-8 values between the two treatment groups are not statistically significant for IL-8 (*p* = 0.93), further highlighting that heat-killed DPC3147 cells have the ability to cause an equally potent localized IL-8 response as viable DPC3147 cells (Figures 1, 2).

### Cure Rates Based on SCC Are Statistically Similar for Treatment Groups Involving Either Viable or Heat-Killed *Lactococcus lactis* DPC3147 Cells

Quarters with a SCC of >250,000 cells/ml (log 5.4 cells/ml), were selected for this study as they were representative of mastitis. Somatic cell counts are an indicator of infection and inflammation in the udder, with a higher SCC generally indicative of inflammation. The criteria for defining chronic mastitis included cows that historically had suffered from recurrent cases of mastitis in the herd prior to the commencement of this current trial, and cows that were on occasion slow to recover from mastitis even with antibiotic therapy. These cows on occasion failed antibiotic therapy and recovered from mastitis naturally as a result of mounting a natural immune reaction to combat the infection. Such historical records were retrospectively checked upon completion of the trial, to prevent bias when initially selecting cows in the herd with high bulk (and high quarter) SCCs, as part of this current trial. The cure rates as part of this current trial were evaluated based on the initial SCC compared to counts after 7 days post-infusion. While a minimum SCC of >250,000 cells/ml (log 5.4 cells/ml) was chosen as a criterion for selecting cows with mastitis, the majority of animals selected had chronic mastitis and starting quarter SCCs which were significantly higher than this minimum criterion value of 250,000 cells/ml. Milk samples were taken from mastitic cows at the following time points: T0 (before treatment), T0.25 (6 h post-infusion), T1, T2, T3, T5, and T7 days. A clinical cure was considered as a reduction in SCC from initially high starting counts to a SCC of between 250,000 and 750,000 cells/ml within 5–7 days post-treatment, as well as the absence of pathogens upon plating (Tables 1 and 2). It was shown that the cure rate of both treatment groups was somewhat similar and there was no statistically significant difference between the groups in terms of cure rate (*L. lactis* DPC3147 viable cells = 46.67% cure rate, *L. lactis* DPC3147 heat-killed cells = 15.38% cure rate), (*p* = 0.2616 when cured numbers were compared to each other and *p* = 0.5556 when non-cured numbers were compared to each other, Fisher’s exact test; Tables 1 and 2). Although there were no statistically significant differences (*p* > 0.05) in the cure rates between the two treatment groups, the percentage cure rates were higher for the live DPC3147 treatment group compared to the heat-killed treatment group. Such a difference in the percentage cure rates is likely to be due to inter-cow variation, which is an inherent part of field trials of this nature. It must also be noted that *S. aureus* was found to be the predominant etiological agent of mastitis in the cows investigated in this trial (Table 1).

**Table 1:** Total number of quarters infused with one of the two emulsion-based formulations, showing the etiological agents of mastitis.

Treatment	Culture-positive cases	Identity of pathogens	Cure rate of culture-positive cases based on SCC results (%)
*L. lactis* DPC3147 live (total *N* = 15 teats)	14/15	12/14 *S. aureus*, 2/14 *Strep. uberis*, 1/14 *Strep. dysgalactiae*	4/12 *S. aureus* (33.33%), 1/2 *Strep. uberis* (50%), 1/1 *Strep. dysgalactiae* (100%)
*L. lactis* DPC3147 heat-killed (total *N* = 13 teats)	9/13	7/9 *S. aureus*, 2/9 *Strep. uberis*	1/7 *S. aureus* (14.29%), 0/2 *Strep. uberis* (0%)

Cows with chronic mastitis were predominantly selected in this trial. *N* number denotes number of quarters infused per treatment group. A clinical cure was defined as a reduction in SCC to 250,000–750,000 cells/ml within 5–7 days of treatment and the absence of pathogens upon plating.

**Table 2:** Overall cure rates for each of the treatment groups investigated in this trial.

Treatment group	*N* (number of quarters)	Cure rate (%)
*L. lactis* DPC3147 live	15	7/15 (46.67%)
*L. lactis* DPC3147 heat-killed	13	2/13 (15.38%)

Cows with chronic mastitis were mainly selected in this trial. A clinical cure was defined as a reduction in SCC to 250,000–750,000 cells/ml within 5–7 days of treatment and the absence of pathogens upon plating.

**Figure 3:**
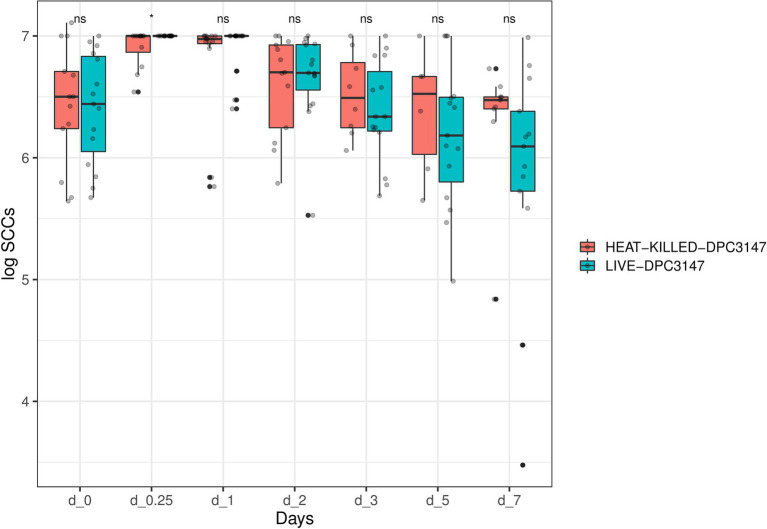
A non-parametric Wilcoxon test was used to compare the SCC values in cows treated with viable DPC3147 cells versus heat-killed DPC3147 cells. There were no statistically significant differences in the SCC values between the two treatment groups at the designated time points (*p* > 0.05 in all cases), with the exception of time point Day 0.25 (6 h post-infusion), where there was a statistically significant difference in the SCC values between viable and heat-killed cells (*p* < 0.05), depicted by an asterisk at Day 0.25. Due to the lack of statistically significant differences at each of the time points except Day 0.25, the data indicates that heat-killed cells may be as efficacious as viable DPC3147 cells in terms of decreasing SCC count in mastitic cows.

Overall, as was the case for IL-8 values, it was determined that the SCC data was not normally distributed, by performing a Shapiro test of normality (*p* < 0.05). Therefore, a non-parametric Wilcoxon test was used to compare the SCC values in cows treated with viable DPC3147 cells versus heat-killed DPC3147 cells (Figure 3). As was the case for the IL-8 values, there were no statistically significant differences in the SCC values between the two treatment groups at the designated time points (*p* > 0.05 in all cases), except for time point Day 0.25 (6 h post-infusion), where there was a statistically significant difference in the SCC values between viable and heat-killed cells (*p* < 0.05), depicted by an asterisk at Day 0.25 (6 h). Due to the lack of statistically significant differences at all the other time points, the data indicate that heat-killed cells may be as efficacious as viable DPC3147 cells in terms of decreasing SCC counts in mastitic cows.

**Figure 4:**
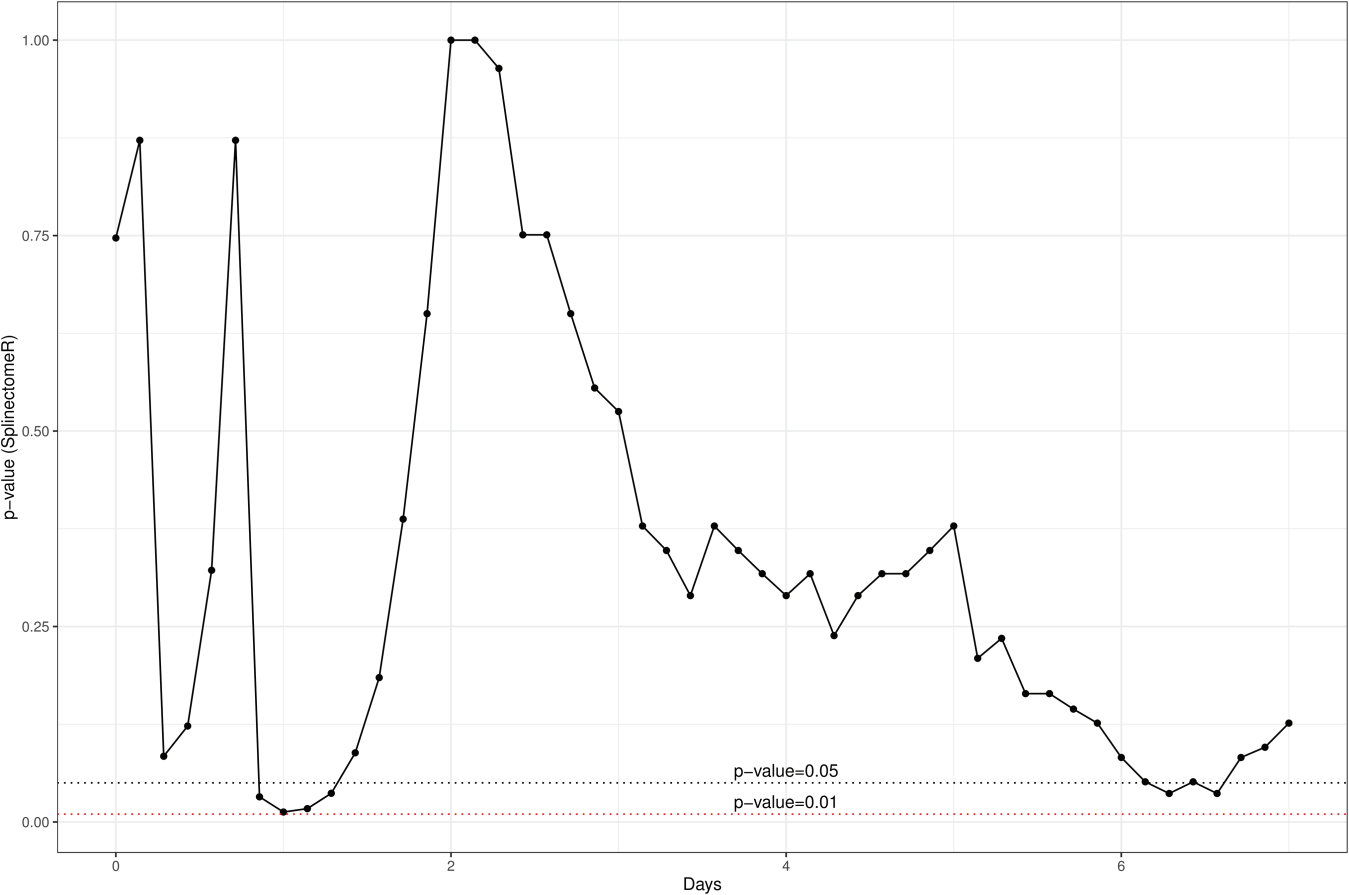
A spline-based statistical tool was to compare the value of *p* of SCC values in the whole time series (R package splinectomeR; Shields-Cutler et al., 2018). Overall, the differences are not statistically significant for SCC between the two groups (*p* = 0.38). However, this spline-based model predicts that the comparison is statistically significant at Day 0.25 (as shown previously in Figure 2) and also statistically significant between the two treatment groups on Day 5 and Day 7 (*p* < 0.01). Overall, this spline-based analysis also indicates that heat-killed DPC3147 cells can be as efficacious as viable DPC3147 cells in terms of decreasing SCC count in mastitic cows.

**Figure 5:**
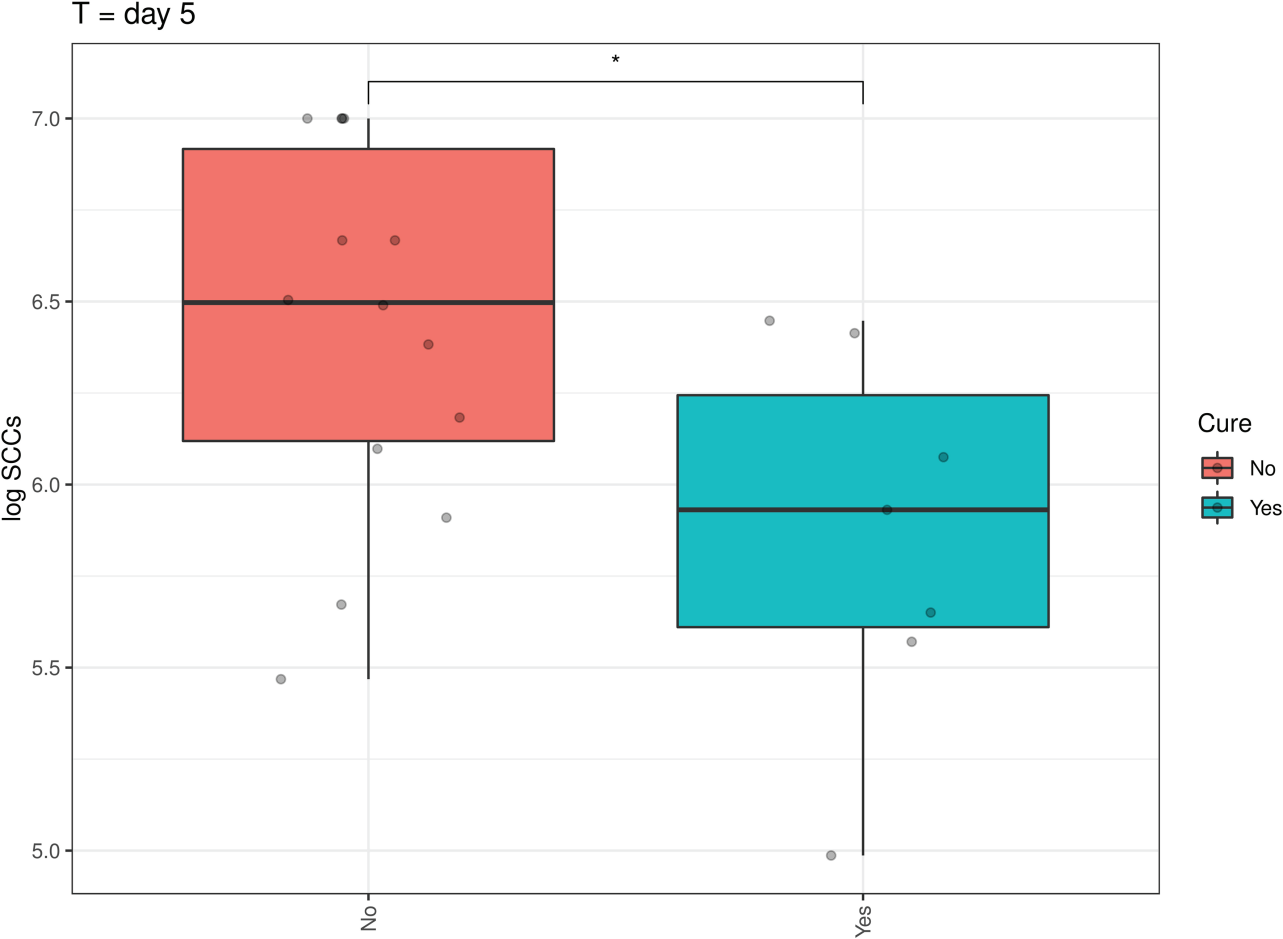
A Wilcoxon comparison in SCC values between cured cows versus non-cured cows was conducted. The Wilcoxon test showed that there was a statistically significant difference in SCC values (*p* < 0.05) between cured cows versus non-cured cows at Day 5 post-infusion, indicating that it may take up to 5 days post-infusion to see clinical differences in the cows in terms of defining a clinical cure.

In addition, a similar spline-based statistical tool as described in Figure 2 was used to compare the value of *p* of SCC values in the whole time series (R package splinectomeR; Shields-Cutler et al., 2018; Figure 4). As briefly mentioned above for Figure 3, overall, the differences in SCC values between the two treatment groups was not statistically significant (*p* = 0.38). However, this spline-based model could predict that the comparison was statistically significant between the two treatment groups at Day 0.25 (as shown previously in Figure 3) and also statistically significant on Day 5 and Day 7 (*p* < 0.01). Overall, this spline-based analysis also indicates that heat-killed DPC3147 cells can be as efficacious as viable DPC3147 cells in terms of decreasing SCC counts in mastitic cows (Figure 4). Furthermore, a Wilcoxon comparison between cured cows versus non-cured cows was conducted. The Wilcoxon test showed that there was a statistically significant difference in SCC values (*p* < 0.05) between cured cows versus non-cured cows at Day 5 post-infusion, indicating that it may take up to 5 days post-infusion to see clinical differences in the cows in terms of defining a clinical cure (Figure 5).

This trial established that the cure rates of both treatment groups were statistically non-significant (with the heat-killed *L. lactis* DPC3147 group showing somewhat lower cure rates, most likely due to inter-animal variation which is common in field trials of this nature). The focus of our previous trial was to compare the efficacies and cure rates of an emulsion-based formulation containing the live-biotherapeutic *L. lactis* DPC3147 versus commercial antibiotics to treat bovine mastitis. It is likely that any resolution and cure of the disease in trials of this nature is predominantly due to the cow’s immune system mediated by IL-8 resulting from infusion with our treatment formulations, rather than the direct antimicrobial activity of lacticin 3147 against the pathogens. This was demonstrated here in this trial, as the cure rates of both treatment groups were statistically comparable to each other (Tables 1 and 2).

### IL-8 Values Are Positively Correlated With SCC Values in Cured Cows but Not in Non-cured Cows

**Figure 6:**
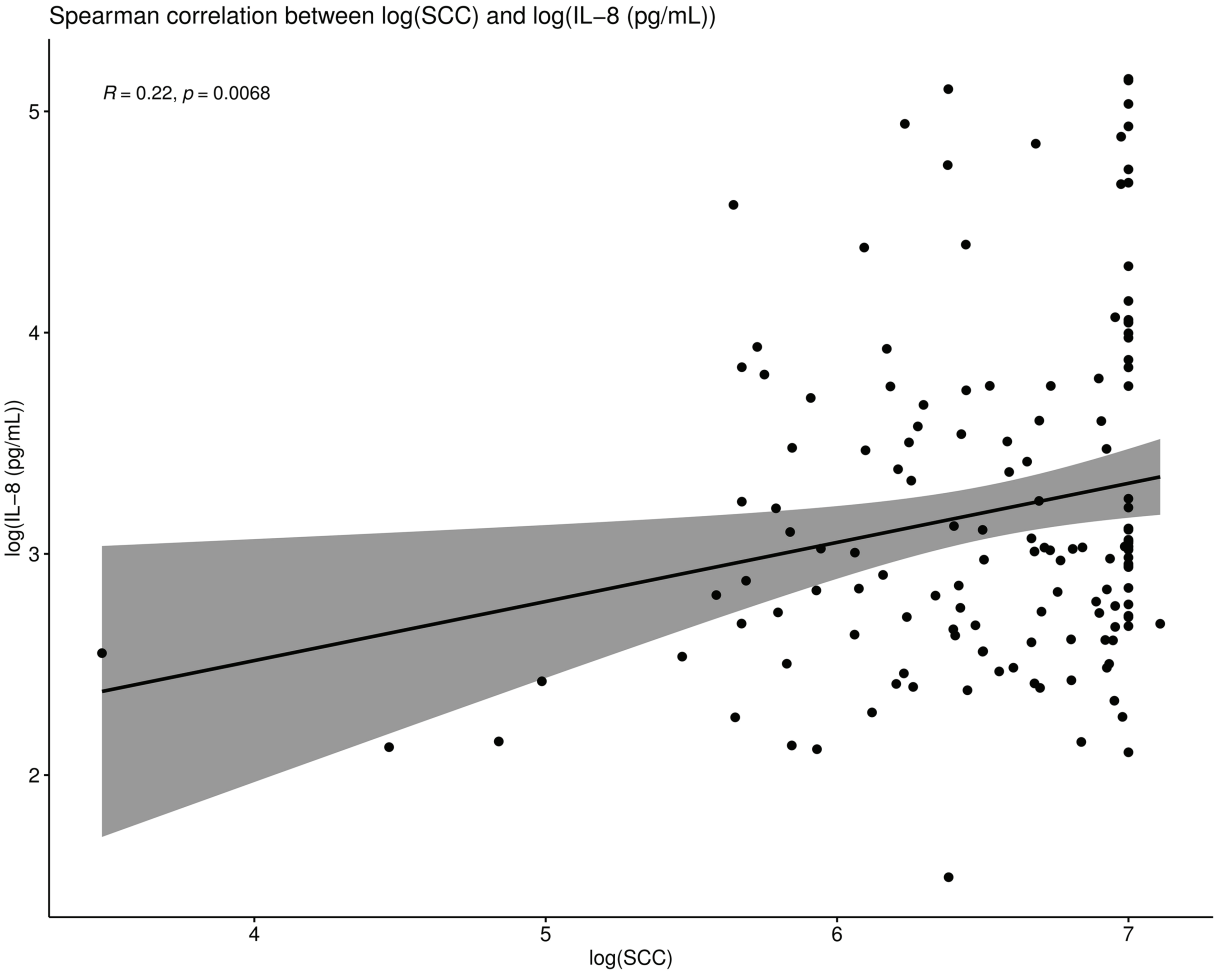
Spearman correlations were conducted between IL-8 values and SCC values. When compared overall, SCC and IL-8 are positively and significantly correlated (*R* = 0.22, *p* < 0.01).

**Figure 7:**
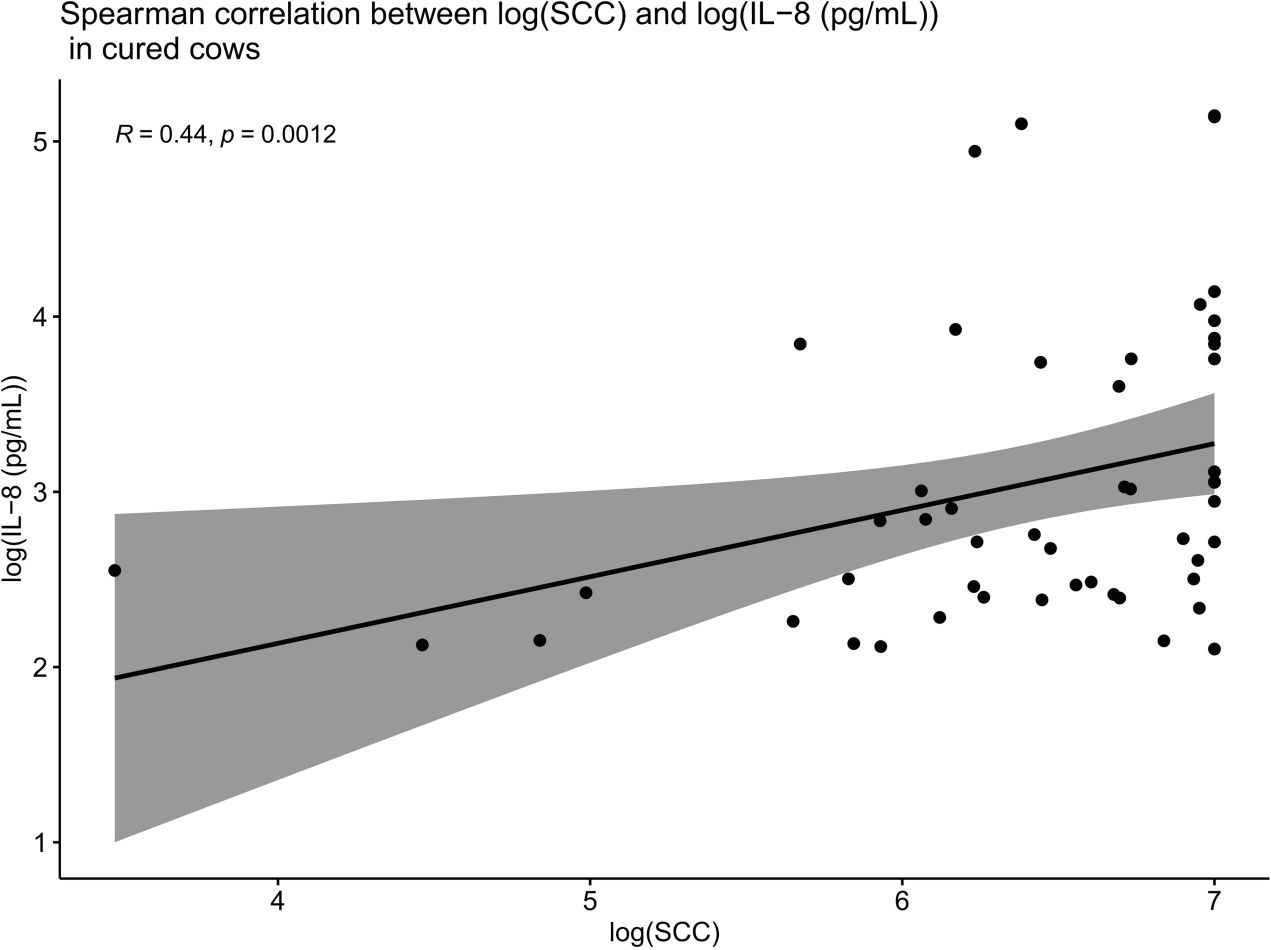
Spearman correlations between IL-8 values and SCC values in cows which were cured showing that when compared overall, SCC and IL-8 are positively and significantly correlated in cured cows (*R* = 0.44, *p* = 0.0012). This suggests that increased IL-8 values in response to infection (and therefore high SCC values) result in the recruitment of PMNs to the site of infection, which likely combat the offending pathogen, eventually resulting in a clinical cure.

Spearman correlations were conducted between all the IL-8 values and SCC values as part of this trial. When compared overall, SCC and IL-8 were positively and significantly correlated (*R* = 0.22, *p* < 0.01; Figure 6). Such Spearman correlations were then conducted between IL-8 values and SCC values in cows which were cured (Figure 7). When compared overall, SCC and IL-8 are positively and significantly correlated in cured cows (*R* = 0.44, *p* = 0.0012). Although the concentrations of PMNs were not measured in this trial, the trends described above suggest that increased IL-8 values in response to infection (and therefore high SCC values) likely resulted in the recruitment of PMNs to the site of infection, which helped combat the offending pathogen, eventually resulting in a clinical cure (Figure 7).

**Figure 8:**
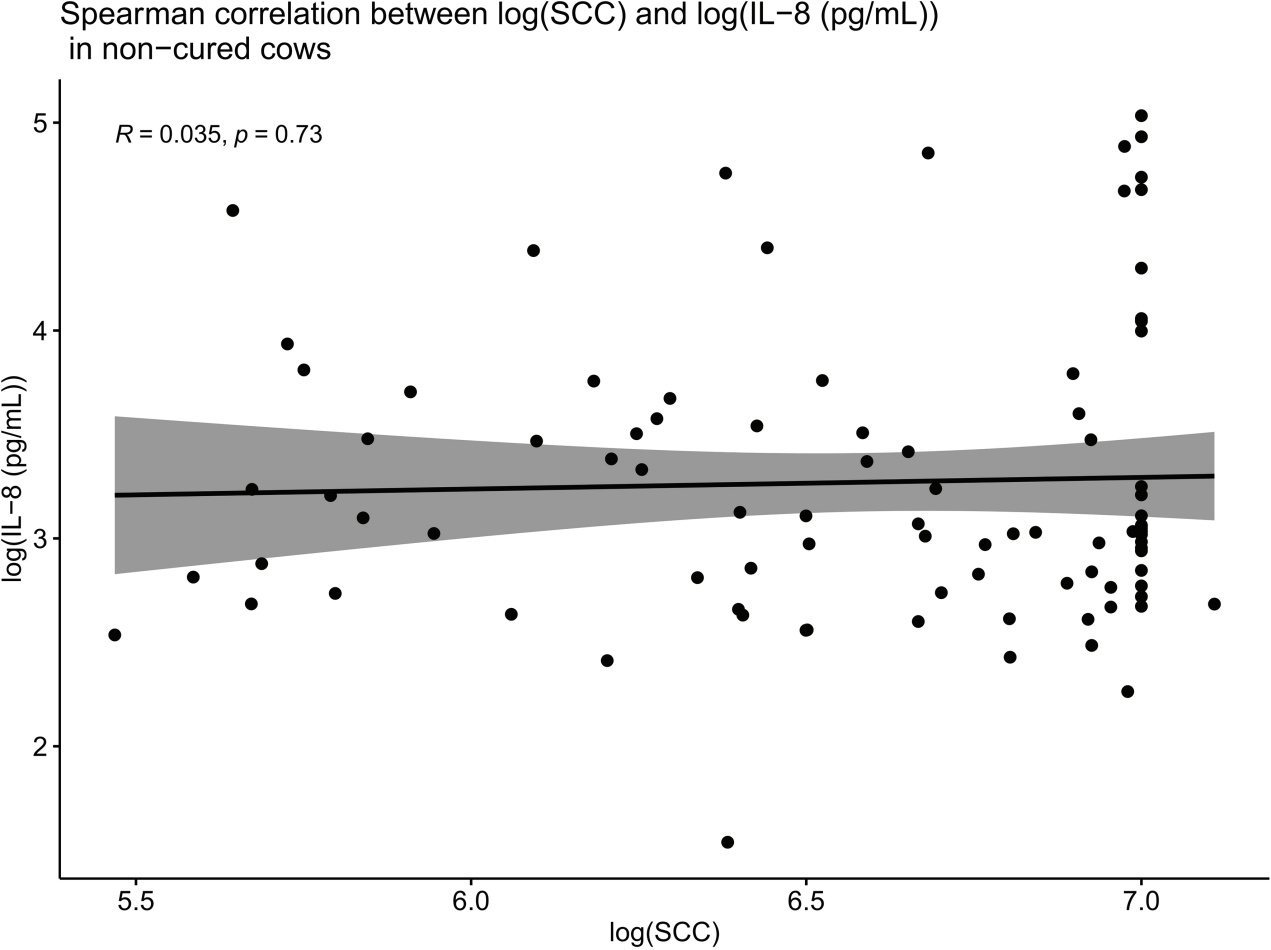
Spearman correlations between IL-8 values and SCC values in cows which were not cured. When compared overall for cows which were not cured, SCC and IL-8 are no longer positively correlated (*R* = 0.035, *p* = 0.73). This suggests that these cows were likely unable to mount a sufficient localized IL-8 response, leading to inadequate recruitment of PMNs to the site of infection. This insufficient localized IL-8 response may explain why such cows were unable to combat the infection and thereby remained uncured.

Such Spearman correlations were also conducted between IL-8 values and SCC values in cows which were not cured (Figure 8). When compared overall for cows which were not cured, SCC and IL-8 were no longer positively correlated (*R* = 0.035, *p* = 0.73). While we do not have data pertaining to PMN concentrations at the site of infection in this trial, the lack of positive correlations between SCC and IL-8 in non-cured cows described above suggests that these cows were likely unable to mount a sufficient localized IL-8 response, leading to inadequate recruitment of PMNs to the site of infection. This insufficient localized IL-8 response may help to explain why such cows may have been unable to combat the infection and thereby remained uncured.

### Cows in This Trial Mounted an Equally Potent Localized IL-8 Immune Response, Irrespective of the Pathogenic Etiological Agent of Mastitis

**Figure 9:**
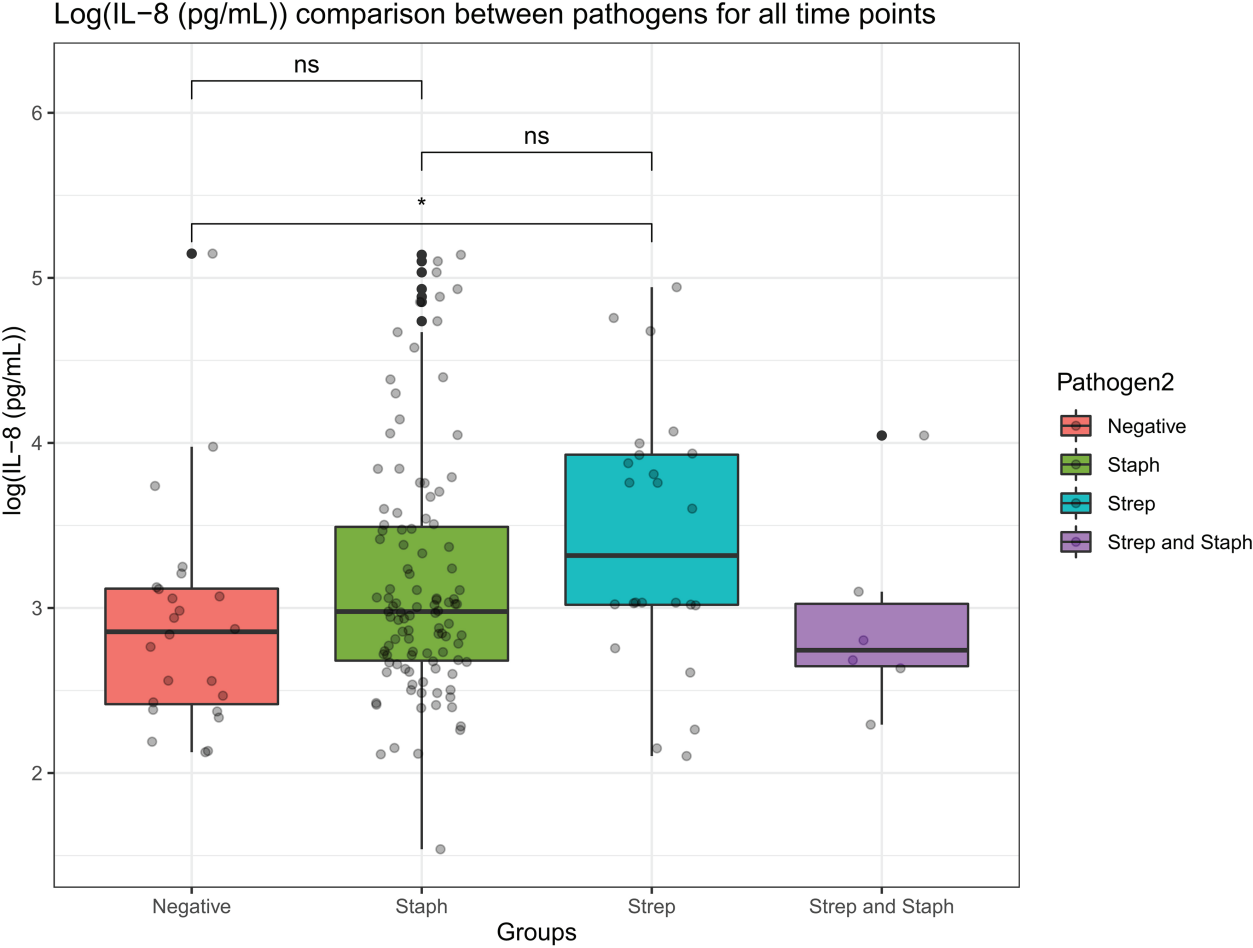
A Wilcoxon comparison was conducted for IL-8 values in cows where the etiological agent of mastitis was identified to be *Staphylococcus*, *Streptococcus*, *Staphylococcus*, and *Streptococcus* together (co-infection) or pathogen negative (where there was no etiological agent detected but the cows still presented mastitis). Overall, the statistical tests show that there were no significant differences in IL-8 values detected in milk samples from the cows infected by each of the above-mentioned pathogens (*p* > 0.05). However, there were statistical differences in IL-8 values detected in the milk samples in cows infected with *Streptococcus* versus cows where there was no pathogen detected (but still suffered from mastitis; *p* < 0.05).

A Wilcoxon comparison was conducted for IL-8 values in cows where the etiological agent of mastitis was identified to be (i) *Staphylococcus aureus*, (ii) *Streptococcus* species, (iii) *Staphylococcus aureus* and *Streptococcus* species together (co-infection), or (iv) pathogen negative (where there was no etiological agent detected but the cows still presented with mastitis). Overall, the statistical tests show that there were no significant differences in IL-8 values detected in milk samples from the cows infected by each of the above-mentioned pathogens (*p* > 0.05; Figure 9). However, there were statistical differences in IL-8 values detected in the milk samples in cows infected with *Streptococcus* species versus cows where there was no pathogen detected (but still suffered from mastitis). The average IL-8 values were higher in *Streptococcus*-infected cows compared to cows where there was no pathogen detected. This may be due to the cows’ natural immune response detecting the *Streptococcus* pathogen in the infected udder and mounting a natural localized IL-8 immune response as a result of detecting the pathogen.

## Discussion

Upon completion of this trial, we now have a more comprehensive hypothesis and a better understanding of the mechanism of action of emulsion-based formulations containing *L. lactis* cells. Prior to the commencement of the trial, we sought to answer one main key question: whether emulsion-based formulations need to contain viable *L. lactis* cells or not, in order to stimulate a localized immune response. Based on the findings of this field trial, we have now established that infusion of emulsion-based formulations containing heat-killed *L. lactis* cells can evoke an equally potent localized IL-8 response in infected udders, as can viable cells. It may be the case that cell surface-associated structures are responsible for inducing a localized IL-8 immune response in cows with mastitis. In addition, differences in the composition of cell surface components between *S. aureus* and *Streptococcus* species may also account for the differences observed in IL-8 production in this trial, as shown in Figure 9. It is likely that any clinical cure is due to a stimulation of the cow’s immune response, triggered by either viable *L. lactis* DPC3147 cells and/or partially degraded components of the cell walls of heat-killed DPC3147 cells. Indeed, there may be a combination of partially intact and partially degraded *L. lactis* cell wall components upon heat treatment. We aim to gain insights into the integrity of the *L. lactis* envelop by electron microscopy and ascertain the role of any such partially degraded or fully intact lactococcal cell wall components as part of future trials. Taken together however, the IL-8 data reported herein in this trial indicate that the immune response may be triggered by some other factor associated with the cell wall, perhaps fragments of peptidoglycan, or teichoic acid associated with *L. lactis* and/or Gram-positive cell walls, rather than being directly associated with viable cells. Indeed, it is highly likely that by heat-treating the *L. lactis* cells, such components of cell wall fragments such as peptidoglycan and teichoic acid are released into the emulsion. The presence of these foreign substances may in turn trigger an immune response in the udder, which likely explains the increase in IL-8 titres.

Previous trials of this nature investigating heat-killed and viable cells have shown that viable cells elicit a more pronounced influx of lymphocytes and neutrophils to the site of infection (Crispie et al., 2008). Unlike some of these previous trials of this nature which focused on various aspects of the immune response, this current trial predominantly focused on a localized IL-8 response at the site of infection upon infusion with viable and heat-killed cells present in emulsion-based formulations, as a previous trial had indicated that this was the main cytokine involved in evoking an immune response and resolving the infection (Beecher et al., 2009). In the trial conducted by Crispie et al. (2008), heat-killed cells evoked a less potent influx of lymphocytes and neutrophils, relative to the viable cells. The mean neutrophil counts increased by 10-fold 1 day post-infusion, and the peak PMN counts occurred on day 3. Although there was a rise in PMN numbers, this was significantly lower than the numbers elicited by viable cells in the trial (Crispie et al., 2008). Infusion with live cultures resulted in the mean PMN values peaking on days 1 and 2 post-infusion. Similarly, the mean lymphocyte levels peaked on day 3 post-infusion. The viable cells evoked significant increases in milk amyloid A (MAA) and haptoglobin (Hp) levels. While heat-killed cells evoked an increase in APP levels, these levels were lower than those evoked by viable cells in that trial (Crispie et al., 2008). The stronger immune response elicited by viable cells in the trial conducted by Crispie et al. (2008), may have accounted for the differences between the clinical cure rates in that trial compared to our current trial. In contrast to the above-mentioned trial, our current trial here predominantly focussed on a localized IL-8 response in cows (the majority of which were affected with chronic cases of mastitis). We found that heat-killed cells present in emulsion-based formulations elicited a potent IL-8 response. However, it remains to be determined whether this increase in IL-8 triggered a substantial enough recruitment of PMNs to the site of infection. An insufficient recruitment of PMNs, below the threshold required to fight the infection, may account for the somewhat low cure rates found in this current study. It must also be emphasized that there was a large inter-animal variation in terms of immune responses in this trial.

While the cure rates in this study are somewhat lower than desired, it is important to note that most cows selected for this field trial suffered from recurrent chronic mastitis and thus were more likely to fail any type of antimicrobial therapy, including commercial antibiotics and/or our two treatment formulations. While we do not have data directly comparing the cure rates of antibiotics versus our two emulsion-based formulations in cows with chronic mastitis as part of this current trial here, we hope to ascertain the effects of antibiotics against such chronic cases of mastitis in these same cows as part of future field trials and compare the efficacy of antibiotics to our emulsion-based formulations containing heat-killed cells, pending ethical approval. In addition, as part of this current trial, *S. aureus* positive cultures were the most frequently seen cause of disease. *Staphylococcus aureus* has also been found to be the most common cause of mastitis in Irish dairy herds overall (Keane et al., 2013). Indeed, the phenotypic and genomic plasticity of *S. aureus* contributes to its persistence. *Staphylococcus aureus* persistence is most commonly linked to their ability to invade epithelial cells, form a biofilm and polysaccharide capsules (Bardiau et al., 2014), all of which contribute to the ability of *S. aureus* to evade the innate and adaptive immune response of the cow, thus persisting in the mammary gland. For instance, the presence of small colony variants (SCVs) in dairy cows with a history of chronic intra-mammary *S. aureus* infection (Atalla et al., 2008) appear to have atypical morphological and biochemical properties, and it can survive in the intracellular environment that protects the bacteria from host defences and antibiotics (Atalla et al., 2011). Furthermore, the synthesis of polysaccharide capsules can stop the action of macrophages, mask the recognition of antibodies directed against the cell wall by PMNs and prevent complement activation (Zaatout et al., 2020). A combination of these evasive mechanisms of *S. aureus* in addition to selecting chronic mastitis cases may explain why the cure rates were relatively low in this study, in comparison to some previous trials. Interestingly, our current trial also showed that the cows mounted an equally potent IL-8 response, irrespective of the etiological pathogen involved (either *S. aureus* or *Streptococcus* species). However, the lower cure rates for *S. aureus*-associated mastitis cases in this trial are likely to be due to the above-mentioned phenomena, even though the IL-8 response mounted was statistically as potent as the response mounted in cows with *Streptococcus*-associated mastitis. Furthermore, with regard to cure rates, it was also interesting to note in this trial that SCC values and IL-8 titres were positively correlated in cured cows but there was no such positive correlation in non-cured cows. This may be due to a higher and more potent IL-8 response mounted in cured cows, and the insufficient IL-8 response mounted in non-cured cows may explain why such cows were unable to combat the infection.

Despite the variations seen in different animal trials of this nature, similar to our current trial, several *in vivo* trials have also demonstrated the efficacy of heat-killed cells in stimulating the immune response and potential probiotic effects of heat-killed cells. In one such study, Warda et al. (2019) prepared a heat-killed preparation of ADR159 containing *Lactobacillus delbrueckii*. The study showed that heat-killed cells and components of the cell wall were able to elicit an immune effect. In a separate study, Wagner et al. (2000) tested the effects of administering heat-inactivated *Lactobacillus casei* and *Lactobacillus acidophilus* to *Candida albicans*-colonized immunosuppressed mice. The authors found that heat-killed probiotics conferred protection against *C. albicans*-induced systemic as well as mucosal candidiasis infection. Piqué et al. (2019) summarized the health-promoting properties of tyndallized probiotics, mainly LAB and bifidobacteria, in a comprehensive review and described how heat-killed probiotic cells are still capable of eliciting immunomodulatory effects. Indeed, after inactivation of bacteria, dead cells can release bacterial components with key immunomodulating effects and with antagonizing properties against pathogens. Different bacterial components, such as lipoteichoic acids, peptidoglycans or exopolysaccharides (EPS), have been proposed to be mainly involved in these properties in preparations containing heat-killed bacteria (Taverniti and Guglielmetti, 2011; Sarkar and Mandal, 2016; Castro-Bravo et al., 2018). Another example includes a study by Jorjão et al. (2015) who reported that heat-killed *Lactobacillus rhamnosus* ATCC7469 cells were able to stimulate mouse macrophages to produce cytokines including IL-4, IL-6, IL-10, IL-12, among others. In other bovine mastitis-related studies, Blanchet et al. (2021) demonstrated the immunomodulatory efficacy of heat-killed *Lactobacillus gasseri* LA806 in the process of impairing *E. coli* and *S. aureus* colonization of bovine mammary epithelial cells (bMEC) in *in vitro* models. A separate study by Fukuyama et al. (2020), also reported the immunomodulatory efficacy of LAB strains in stimulating Toll-like receptor-mediated inflammatory responses in *in vitro* bMEC models. Finally, Seridan Assis et al. (2015), demonstrated the efficacy of *L. lactis* V7 in stimulating the production of IL-8 in bMEC cell culture and by doing so, preventing the internalization of *S. aureus* and *E. coli* pathogenic strains into bMEC cells.

While we have gained some further insights into the localized immune response that is elicited by our emulsion-based formulations containing *L. lactis* DPC3147 (and heat-killed cells thereof), this trial does have some limitations, and this is largely due to the limited number of animals (maximum *n* = 15 animals per treatment group) and limited treatment groups one can use in a trial, in keeping with the principles of the 3Rs. Although the inclusion of an antibiotic treatment group as a supplementary control treatment group may have been beneficial as part of this trial, the primary focus of this trial was to answer one main question: whether heat-killed *L. lactis* cells are as efficacious as viable *L. lactis* cells in evoking a localized IL-8 immune response in udders affected by mastitis. Indeed, upon completion of this trial, we now have greater insights into the immune response to heat-killed *L. lactis* cells.

It is anticipated that further trials of this nature with a few other treatment groups may have to be conducted to fully unravel the complex mechanisms involved in the resolution of this disease, pending approval by local animal ethics committees. Future trials of this nature may involve alternative *L. lactis* and/or other LAB strains which do not produce any bacteriocins/antimicrobials at all. A supplementary negative control treatment group may involve infusions with merely the emulsion-based formulation without any cells (live or heat-killed) whatsoever. This would provide insights as to whether the liquid paraffin-Tween 80 based emulsion-associated components trigger a minor immune response on their own and would enable us to quantify localized baseline IL-8 titres at the site of infection in the affected udder. In addition, as heat-killing cells is a very severe procedure, significantly degrading the bacterial cell wall and likely affecting heat-labile components which may have potential immunostimulatory activity, alternative methods such as UV irradiation could also be tested to determine what effect UV-inactivated cells with an intact cell wall have on the immune system. As UV irradiation damages the DNA inside bacterial cells and does not have a severe effect on the cell surface components, it may be the case that UV-inactivated cells are still capable of evoking a strong immune response. A separate negative control treatment group may include mastitic cows which have not been infused with any foreign substances at all after the initial detection of mastitis, in order to determine the strength of the natural immune response and baseline IL-8 titres evoked by the immune system of the cow itself in response to the pathogen. However, such a negative control treatment group would require us to treat the cows as soon as possible in the event of exacerbating mastitis, in accordance with good veterinary practice. Nonetheless, possible means to circumvent such issues may include a significantly reduced number of cows in such a negative treatment group and withholding treatment with antibiotics or other bio-therapeutic options for the shortest period of time possible. Finally, another possible study could involve assessing the impact of each of these treatment formulations on the microbiome of the teat canal and whether such alterations in the microbiome confer protection against mastitis-causing pathogens or not.

In addition, as part of future trials of a similar nature (pending ethical approval), we hope to quantify the concentrations of lacticin 3147 that are excreted in the milk samples at each of the time points post-infusion to use as an estimate of the amount of lacticin 3147 produced in *in vivo* conditions. Our working hypothesis is that *L. lactis* DPC3147 cells may not be able to produce sufficient quantities of lacticin 3147 in these drastic *in vivo* conditions (and/or cells produce lacticin 3147 at sub-inhibitory concentrations, much lower than the minimum inhibitory concentrations actually required to have a killing effect on the offending pathogen). We aim to incorporate such lacticin 3147 concentration measurements as part of future trials of a similar nature, perhaps by including RP-HPLC and area under the curve analysis to quantify the concentrations of lacticin 3147 which are excreted in the milk samples post-infusion and use such concentrations as an estimate to gain insights into the concentrations produced *in vivo*. Also, further trials of this nature will help to determine whether other lactococci and/or other LAB strains in general, can mount an equally potent localized IL-8 response. Another interesting comparison would be to determine the difference in the potency of an immune response following infusions with other Gram-positive versus Gram-negative strains. However, for such a comparison to be conducted, the strain of choice would have to be carefully selected, ensuring that any such strains used have acquired GRAS and Qualified Presumption of Safety (QPS) status, and pass all the required regulatory safety assessments. Nonetheless, overall, the results of the trial reported here indicate that emulsion-based formulations containing heat-killed cells of *L. lactis* DPC3147 (a postbiotic) have the potential to sufficiently evoke a localized immune response in mastitic cows similar to that observed with the live strain. Furthermore, any such formulations containing heat-killed cells would significantly prolong the shelf life of such products compared to equivalent formulations requiring active viable cells, which would have a limited shelf life.

## Data Availability Statement

The raw data supporting the conclusions of this article will be made available by the authors, without undue reservation.

## Ethics Statement

The animal study was reviewed and approved by this field trial was approved by the Teagasc Animal Ethics Committee (Approval number TAEC186-2018) and by the Health Products Regulatory Authority (HPRA; Project Authorization number AE19132/P086).

## Author Contributions

HM and KL are researchers in Moorepark Food Research Centre, Teagasc and drafted the manuscript. HM prepared the various emulsion-based formulations for infusions, associated microbiological work, and ELISAs to quantify IL-8 titres. KL also conducted ELISAs to quantify IL-8 concentrations. JF conducted the SCC determinations and plating of pathogens. NB performed the infusions. GG performed the statistical analyses. JF, NB, PD, MC, CH, CS, and RR revised the manuscript. All authors contributed to the article and approved the submitted version.

## Funding

HM is a researcher in Teagasc Food Research Centre, and this work was funded by the Science Foundation of Ireland (SFI)-funded Centre for Science, Engineering and Technology and the APC grant number SFI/12/RC/2273. Research in CH, CS, and RR laboratories is supported by the Science Foundation of Ireland (SFI)-funded Centre for Science, Engineering and Technology and APC Microbiome Ireland.

## Conflict of Interest

The authors declare that the research was conducted in the absence of any commercial or financial relationships that could be construed as a potential conflict of interest.

## Publisher’s Note

All claims expressed in this article are solely those of the authors and do not necessarily represent those of their affiliated organizations, or those of the publisher, the editors and the reviewers. Any product that may be evaluated in this article, or claim that may be made by its manufacturer, is not guaranteed or endorsed by the publisher.

## Abbreviations

AMR: Antimicrobial resistance
SCC: Somatic cell counts
PMN: Polymorphonuclear neutrophils/leucocytes
GRAS: Generally regarded as safe
QPS: Qualified presumption of safety
PBS: Phosphate-buffered saline
MRD: Maximum recovery diluent
IL-8: Interleukin 8
ELISA: Enzyme-linked immunosorbent assay
